# High-resolution disease maps for cancer control in low-resource settings: A spatial analysis of cervical cancer incidence in Kampala, Uganda

**DOI:** 10.7189/jogh.12.04032

**Published:** 2022-04-23

**Authors:** Kirsten Beyer, Simon Kasasa, Ronald Anguzu, Robert Lukande, Sarah Nambooze, Phoebe M Amulen, Yuhong Zhou, Brendah Nansereko, Courtney Jankowski, Tonny Oyana, Danielle Savino, Kavanya Feustel, Henry Wabinga

**Affiliations:** 1 Medical College of Wisconsin, Milwaukee, Wisconsin, USA; 2 Makerere University, Kampala, Uganda; 3 Kampala Cancer Registry, Kampala, Uganda

## Abstract

**Background:**

The global burden of cervical cancer is concentrated in low-and middle-income countries (LMICs), with the greatest burden in Africa. Targeting limited resources to populations with the greatest need to maximize impact is essential. The objectives of this study were to geocode cervical cancer data from a population-based cancer registry in Kampala, Uganda, to create high-resolution disease maps for cervical cancer prevention and control planning, and to share lessons learned to optimize efforts in other low-resource settings.

**Methods:**

Kampala Cancer Registry records for cervical cancer diagnoses between 2008 and 2015 were updated to include geographies of residence at diagnosis. Population data by age and sex for 2014 was obtained from the Uganda Bureau of Statistics. Indirectly age-standardized incidence ratios were calculated for sub-counties and estimated continuously across the study area using parish level data.

**Results:**

Overall, among 1873 records, 89.6% included a valid sub-county and 89.2% included a valid parish name. Maps revealed specific areas of high cervical cancer incidence in the region, with significant variation within sub-counties, highlighting the importance of high-resolution spatial detail.

**Conclusions:**

Population-based cancer registry data and geospatial mapping can be used in low-resource settings to support cancer prevention and control efforts, and to create the potential for research examining geographic factors that influence cancer outcomes. It is essential to support LMIC cancer registries to maximize the benefits from the use of limited cancer control resources.

The global burden of cervical cancer is heavily concentrated in low- and middle-income countries (LMICs), having the highest rates in sub-Saharan Africa (SSA) [1]. Cervical cancer is the second most prevalent cancer in LMICs, with approximately 90% of all cervical cancer deaths worldwide occurring in LMICs [2]. Cervical cancer kills more women than other cancers in 28 countries, with 22 of these countries in SSA [1]. A recent study estimated an average 5-year relative survival from cervical cancer in 11 countries in SSA at only 33.1% [3]. Nearly all cervical cancer cases are attributable to Human Papillomavirus (HPV) infection [4], with the highest HPV rates occurring in SSA at 24% [5].

These high cervical cancer incidence and mortality rates occur despite the existence of effective prevention, screening, and treatment methods commonly used in higher resourced settings [6]. Cervical cancer prevention and control requires a comprehensive approach, including vaccination against and screening for HPV (primary prevention), screening and treatment of pre-cancerous lesions (secondary prevention), diagnosis, and treatment of invasive cervical cancer (tertiary prevention), and palliative care. Unfortunately, achieving goals for cervical cancer prevention and control in SSA has proven challenging, largely due to limited resources.

Uganda in eastern Africa ranks seventh in the world for cervical cancer incidence, with an estimated rate of 56.2 per 100 000 people in 2020 (compared to a global rate of 13.3) [7]. Cervical cancer is the leading cause of cancer morbidity and mortality in Ugandan women with an estimated 6959 new cases and 4607 deaths in 2020 [7]. HPV vaccination rates remain low, with approximately 78% of girls unvaccinated in 2016 [8]. Cervical cancer screening rates are low, between 4.8%-30% among eligible women [9-11]. Treatment and palliative care options are also limited for patients with advanced cervical cancer [12]. While Uganda had only one radiotherapy machine serving the country and the region for some time [11,13], there are now three functional external beam radiotherapy machines at the Uganda Cancer Institute – an improvement, though the unmet demand remains high.

In resource-limited settings, targeting scarce resources to populations with the greatest need is critical to maximizing impact. Understanding with specificity *where* cancer burdens are concentrated has the potential to greatly enhance the efficiency of cancer prevention and control efforts. Geospatial mapping of population-based cancer registry data, used commonly in high-resource settings, has not been widely used in LMICs [14], with notable exceptions, primarily outside of SSA [15-20]. High-resolution cancer maps could provide clear targets for cancer prevention and control efforts in SSA and other low-resource settings, optimizing service delivery for maximal benefit.

The objectives of this study were to geocode cervical cancer data from a population-based cancer registry in Kampala, Uganda, to create high-resolution disease maps for potential use in cervical cancer prevention and control planning, and to share lessons learned to strengthen future efforts in Uganda and other low-resource settings. The implications of these findings for cervical cancer prevention and control are discussed.

## METHODS

### Study setting

**Figure 1:**
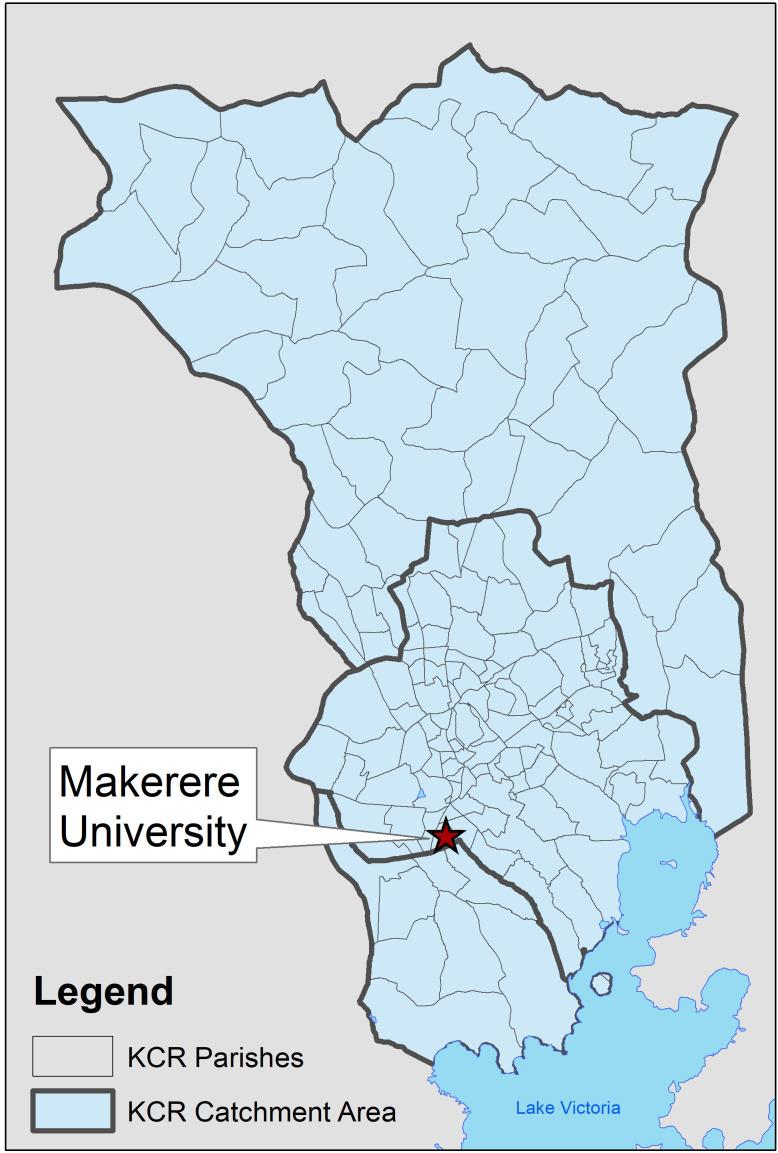
Kampala Cancer Registry catchment area.

The catchment area of the Kampala Cancer Registry (KCR) covers the population of Kyadondo County in Central Uganda, which includes Kampala Capital City Authority (KCCA) and part of Wakiso district (**Figure 1**). The population is predominantly Ganda from the Bantu ethnic group. The study area includes 11 sub-counties containing 146 parishes. The KCR office is located at the Makerere University, Mulago Hospital Complex in the centre of the region. With regard to cancer care in the region, Mulago National Referral Hospital located in Kawempe Division in Kampala District provides chemotherapy at the Uganda Cancer Institute while radiotherapy services are offered through the Department of Radiotherapy. Histology services in Kyadondo county are provided through two histopathology departments in Mulago Hospital and three private histopathological laboratories (Metromed, MultiSystems and Surgipath). In addition, there are three other missionary hospitals and a hospice facility that also provide cancer treatment services.

### Kampala Cancer Registry structure and operations

The Kampala Cancer Registry (KCR) is housed in the Department of Pathology at the Makerere University College of Health Sciences and has the longest time series of cancer incidence in Africa, with records dating back to the 1950s. Cancer is not a notifiable or reportable disease in Uganda, and therefore KCR carries out active surveillance. This involves cancer registrars regularly visiting multiple data sources, including hospitals, hospices, and histopathology laboratories. Within these units, and with the help of designated staff in the records departments with retrieving clinical files, the registrars peruse these files, inpatient and outpatient registers and pathology reports. Cases with a diagnosis of cancer are identified and data from those diagnosed while residents of Kyadondo County are abstracted onto notification forms. Place of residence is defined as the place the patient resided over the last 12 months. Patient residence at diagnosis is obtained from records across the different data sources. KCR reviews all records, including notes and referral letters, in order to identify the earliest residence corresponding to the date of diagnosis and confirm that the patient resided in the catchment area at diagnosis. Data are then entered into the registry database using the CanReg system, which at this stage of data entry prevents the use of non-valid codes and performs checks for internal consistency among variables. The system also identifies potential duplicates and identified records that are completed, corrected, or deleted after tracing the original archives in these various multiple sources.

### Data sources

KCR tumour registry records were used to measure cervical cancer incidence. KCR data are commonly collected via paper forms from local health care facilities and laboratories, which are then entered and stored electronically. However, information on patient residence has not previously been available electronically, although it has been collected in hard copy format. The CanReg database was modified to create new variables for geographic units, aligned with Uganda Bureau of Statistics (UBOS) 2014 geographies [21]. Data for district, sub-county, and parish of residence at diagnosis for cervical cancer was abstracted from KCR paper forms and entered electronically. Not all paper forms included complete geographical information. In cases where the parish of residence was available, but other geographies were not, KCR staff used internet searches (ie, Google Maps) and an online land conflict map tool (http://www.lcmt.org/uganda) developed by the Ministry of Lands in Uganda to identify the corresponding larger geographies: sub-county and district. Data entered were limited to diagnosis dates between 2008 to 2015 (n = 1906). Age and sex-stratified census data for parish and sub-county populations from 2014 (the most recent Ugandan census) for the Kampala and Wakiso districts comprising Kyadondo county were obtained from the Uganda Bureau of Statistics (UBOS) and used to calculate cervical cancer incidence ratios.

### Data cleaning and preparation

The data abstracted from KCR paper records were examined and cleaned to ensure data quality. Abstracted data were imported into STATA 15/SE (StataCorp, College Station, TX, USA). Data were explored and checked for errors. Among 1906 records, 33 records were identified that fell outside of the KCR catchment area and were excluded from the sample. Among the remaining 1873 records, 89.6% (n = 1678) of records included a valid sub-county and 89.2% (n = 1671) of records contained a valid parish. 16 records had an unknown age. There were seven records with valid sub-county but invalid parish data; given the small number of records, and to ensure maps were comparable across geographic units used, we used one data set based on parish completeness for the analyses. The final analytical data set included a total of 1655 records.

### Data analysis

KCR data were summarized using descriptive statistics to describe the cohort. The cervical cancer Standardized Incidence Ratio (SIR) was estimated for each sub-county and as a continuously defined surface, based on parish-level data. Indirect age adjustment was selected instead of direct age adjustment, as is common in geospatial studies, due to small local populations resulting in unstable local rates. For the sub-county analysis, ratios (observed/expected) were calculated and then joined to sub-county boundaries. They were later marked using a red-to-blue divergent colour scheme. Observed cases at the sub-county level were divided by the expected number of cases for each sub-county, which was obtained by multiplying the overall catchment area age-specific rates with the number of people in each age group in each sub-county. Six age categories were used: 20-29, 30-39, 40-49, 50-59, 60-69, and ≥70 years. Sub-county ratios were joined to sub-county boundaries and marked using a red-to-blue divergent colour scheme. Then, to create a continuous surface map, the SIR (observed/expected) was again calculated, this time based on parish-level data. The adaptive spatial filtering (ASF) disease mapping method was used. In ASF, a grid of estimation points is placed across the study area and a ratio is estimated at each grid point by pulling in data from multiple nearby locations (parish centroids) until a pre-defined threshold is met. This approach maximizes geographic detail while ensuring statistical stability of calculations and protecting data confidentiality [22]. ASF was implemented using R software. A uniform, one-kilometre grid was used to estimate ratios across the map. We set thresholds for defining filters at 20, 25 and 30 expected cervical cancer cases to explore the influence of the threshold on resulting spatial patterns. A final threshold value of 30 expected cases was selected. Observed and expected numbers were calculated and located at each parish centroid. ArcGIS 10.6 was used to interpolate surfaces using inverse distance weighting based on estimated cervical cancer standardized incidence ratios for each grid point to generate a continuously defined surface representing age-adjusted cervical cancer incidence across the study area. The continuously defined map was additionally imported into Google Earth with 50% opacity to facilitate the interpretation of identified patterns. Pertinent locations were also identified in Google Earth and included in visualizations to enhance interpretation.

### Ethics approval

This work was reviewed and approved by the ethics boards at the Medical College of Wisconsin and Makerere University and approved by the Uganda National Council on Science and Technology.

## RESULTS

**Table 1** summarizes the characteristics of the cohort; it includes 1655 women diagnosed with cervical cancer between 2008-2015, with approximately 150-280 women diagnosed each year. Recent years have had higher numbers of cases, with the largest number of diagnoses in 2012 and 2014. The cohort includes women aged 20-93 at diagnosis, with the largest percentage diagnosed at age 40-49. Tumour staging is limited by resource constraints and is not comprehensive, with 54% missing stage information. Vital status was identified as deceased for 13.6% of the population, but this number is likely an undercount, given the limited resources for regular follow-up to confirm vital status. Numerous tribes were identified; only those with 50 or more cases are listed in the table. The Baganda tribe comprised 57.5% of all cases.

**Table 1:** Characteristics of cervical cancer cohort in the KCR catchment area (n = 1655)

Characteristic	Count	Percent
**Year of diagnosis:**		
2008	148	8.9
2009	150	9.1
2010	171	10.3
2011	211	12.8
2012	278	16.8
2013	210	12.7
2014	267	16.1
2015	220	13.3
**Age group (years):**		
20-29	127	7.7
30-39	403	24.3
40-49	467	28.2
50-59	343	20.7
60-69	188	11.4
70 and above	127	7.7
**Stage at diagnosis:**		
1 Localized	69	4.2
2 Local spread	204	12.3
3 Regional spread	311	18.8
4 Advanced, distance metastasis	86	5.2
9 Stage not determined	87	5.3
Missing	898	54.2
**Vital status:**		
Alive	1253	75.7
Deceased	255	13.6
Invalid code	177	10.7
**Tribes:**		
Baganda	951	57.5
Banyankore/Hima	120	7.2
Banyarwanda	73	4.4
Basoga	59	3.6
Other	381	23.0
Unknown	71	4.3

**Figure 2:**
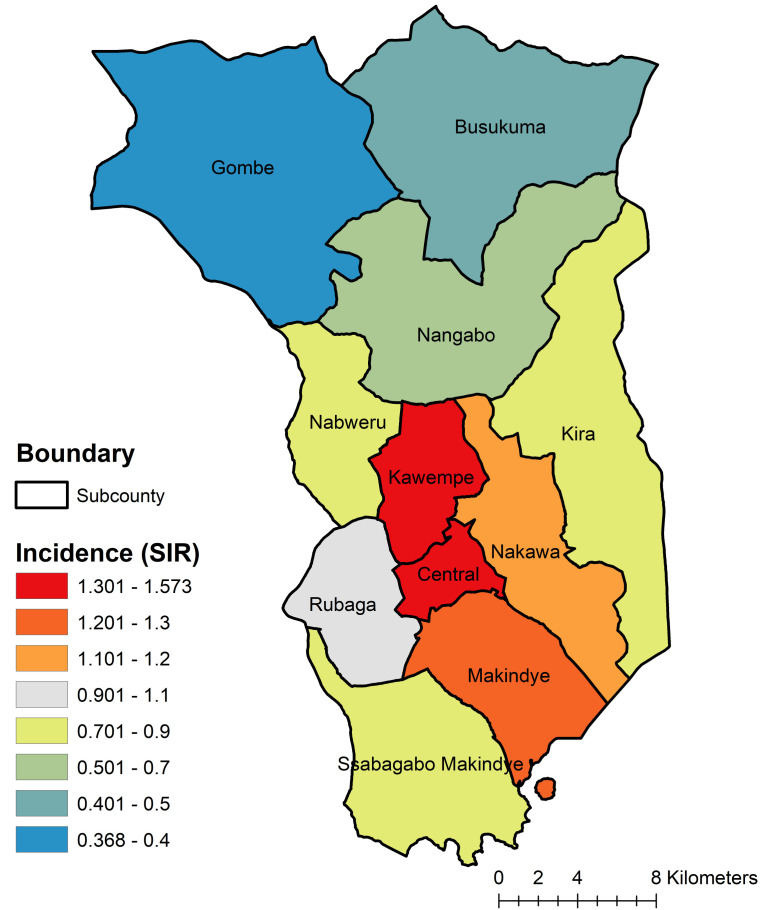
Cervical Cancer Incidence by Subcounty, Kampala Cancer Registry Catchment Area, Uganda, 2008-2015.

A map of cervical cancer incidence by sub-county is displayed in **Figure 2**. The map reveals higher-than-expected cervical cancer incidence in Kawempe and Central sub-counties, with the lowest burden in Gombe sub-county, in the northwestern part of the catchment area.

**Figure 3:**
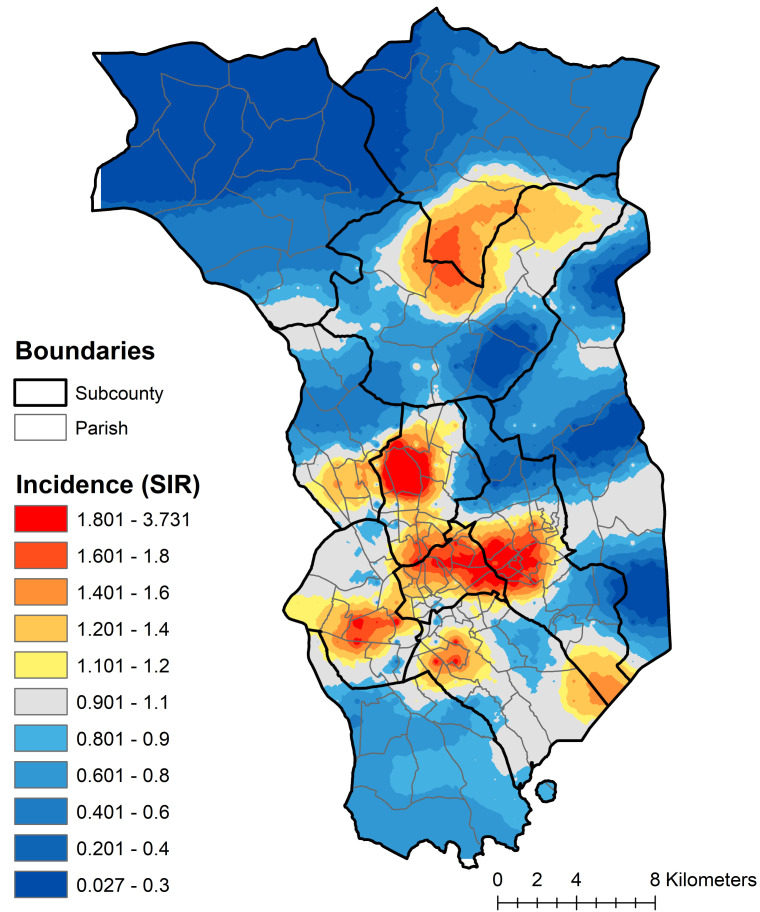
Cervical cancer incidence across the KCR Catchment area, spatially filtered using parish level data, 2008-2015.

A continuously defined map of cervical cancer incidence estimated using parish-level data is displayed in **Figure 3**. Cervical cancer incidence is not evenly distributed within sub-counties; there are patterns of higher- and lower-than-expected incidence within multiple sub-counties, offering evidence to guide the detailed targeting of interventions to at-risk populations.

**Figure 4:**
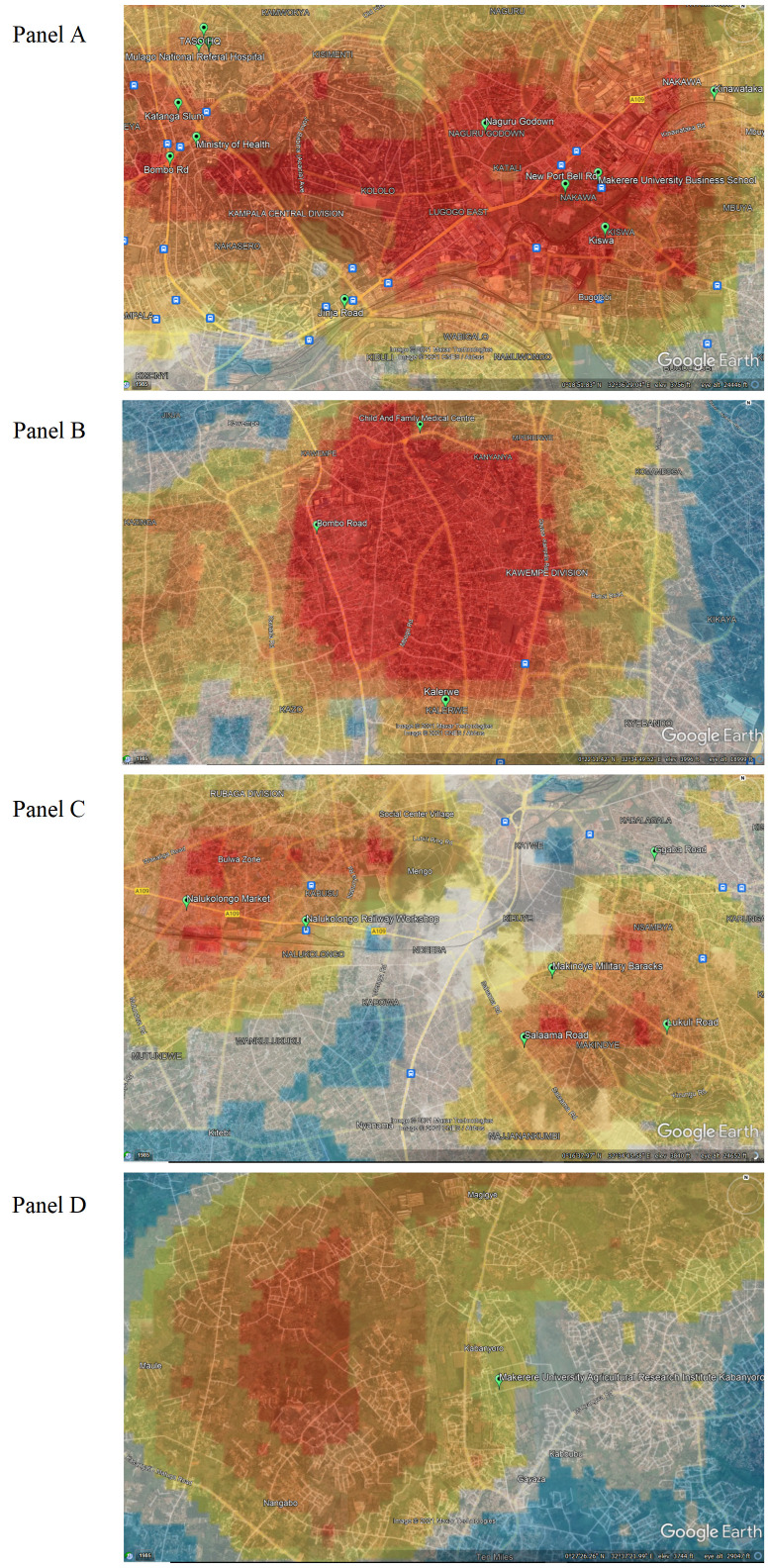
Cervical cancer incidence rates overlaid in Google Earth. Panel A. Kampala Central Division. Panel B. Kawempe Division. Panel C. Rubaga Division. Panel D. Nangabo Division.

**Figure 4****,** Panel A, shows a higher-than-expected incidence that extends horizontally through the parishes of the Kawempe and Central sub-counties, eastward to the parishes of the Nakawa sub-county. On the western edge of the high-incidence area are key resources located in Kawempe sub-county, including the Uganda Cancer Institute, Ministry of Health, Mulago Hospital, and The AIDS Support Organization (TASO) – TASO being especially important, given the comorbidity between HIV and HPV. Also on the western side is the Katanga slum, nestled near Makerere University in Kawempe sub-county. Crossing into Nakawa sub-county, there is a higher-than-expected cervical cancer burden observed on either side of Jinja Road, a major highway and trading route that connects Kampala to Jinja District. High incidence is observed in the Naguru go-down, which is a large slum including low-income households. Moving eastwards, high cervical cancer incidence ratios are observed in Kinawataka slum, another informal settlement of low-income households located west of Kinawataka road and south of Jinja Road in Nakawa sub-county. The Kiswa slum, located south of the New Port Bell Road, also has a high cervical cancer incidence. The Kiswa and Kinawataka slums are located on the south and northeastern side of Makerere University Business School in Nakawa sub-county, respectively.

**Figure 4****,** Panel B, shows high incidence north of the Kampala Northern Bypass Highway, east of Bombo road, and south of the Child and Family Medical Centre. The Kalerwe slum has a higher-than-expected burden of cervical cancer; it is a large, densely populated, informal settlement with urban poor populations. Bombo road is the main trading highway between Kampala and the Northern region of Uganda.

In **Figure 4****,** Panel C, two high incidence ratio areas are found in Rubaga and Makindye sub-counties. Hotspots are seen in the Bulwa zone in Rubaga sub-county, which is an industrial area. Commercial activities include small-scale industries, a large Nalukolongo market, and the Nalukolongo railway hub, suggesting a high turnover of transiting and resident populations in surrounding areas. In Makindye sub-county, cervical cancer incidence is high in the area east of Salaama road and the Makindye Military barracks, on both sides of Lukuli road, south of Ggaba road.

In **Figure 4****,** Panel D, there is high cervical cancer incidence in the northern part of the KCR catchment area, north of the Kasangati-Matugga road located in Nangabo sub-county, and west of the Kabanyoro Agricultural Research Institute of Makerere University.

## DISCUSSION

This manuscript presents the first high-resolution maps of cancer in sub-Saharan Africa using data produced from an African population-based cancer registry. This analysis reveals areas of higher-than-expected cervical cancer incidence that are potential targets for prevention and control activities. Some of these areas are located, fortuitously, in direct proximity to potentially important partners in reducing the cancer burden, including Makerere University, Mulago Hospital, local government offices, and the Child and Family Foundation of Uganda clinic.

Kawempe sub-county has the highest cervical cancer incidence ratio and contains areas of high burden when analysing parish-level data. Kawempe is centrally located and is the most densely populated administrative division in Kampala, with a total population of 338 665 [21]. Kawempe is comprised of a large business hub with large local markets supplied by several road networks [23]. Several large informal settlements like the Katanga slum in Kawempe are associated with poor quality of life and are characterized by their poor housing conditions and high poverty [24]. Kawempe is also affected by a high prevalence of HIV/AIDS, tuberculosis, and their risk factors for transmission [24], which may also be related to HPV prevalence and cervical cancer risk.

According to Uganda’s Ministry of Health, Kawempe has an elevated risk of sexually transmitted infections and a higher incidence of HIV compared to other divisions. As HPV is a sexually communicable virus, patterns of sexually transmitted infections may correspond to patterns of cervical cancer. Commercial sex work is known to be common and frequent among socializing populations in sites located in Kawempe compared to the general population [25]. One study revealed that 75% of 227 hotspots for risky behaviours such as commercial sex work and substance abuse were located in Kawempe [25]. Similarly, 19.2% of men and 12.8% of women reported having paid and received payment for sex with at least one of their most recent three partners [25]. In addition, Kawempe has a high student population, as it includes the Makerere University Kampala (MUK), and in one survey of five large universities in Kampala, a high proportion of female students had chlamydia infection (2.5%) and syphilis (1.7%), while 0.8% and 4.3% of male students had chlamydia and syphilis infections, respectively [26].

The spatially detailed cervical cancer maps presented can be used to advance clinical practice and public health planning efforts to prevent and control cervical cancer in the Kampala, Uganda region. Maps can help to identify high-risk locations and populations to target emergent, innovative, cost-effective interventions for cervical cancer control such as pocket-sized, speculum-free colposcopes [27,28], improved screening colposcopy techniques [29], and community-based HPV self-testing [30].

### Limitations

This study is subject to several limitations. First, this is the first time geographic data collected by the KCR was used. Because the geographic information had not previously been used, it was not a priority for data entry, which resulted in geographical data omission and error. We have taken steps to enhance the quality of geographic information included in the KCR database. A map of parish and sub-county locations, based on UBOS 2014 boundaries, is now used as the reference map for entering geographic data into the database. The paper form used to enter KCR data has been modified to separate geographies of interest into separate fields, limiting error. Further, we have modified the CanReg software data entry interface to allow for direct electronic entry of geographic data into distinct fields for district, sub-county, parish, and village by registry staff, laying the foundation for high-quality geospatial analyses on multiple cancer types in the future. We are actively seeking opportunities to support and enhance the operations of the KCR so as to enable high-quality and timely geospatial analysis in the future. In addition, while KCR makes every attempt to identify the residential address at the time of diagnosis when registering cases, the recorded residence at diagnosis is subject to potential misclassification. It is common in low-resource settings for individuals to come and stay near health care facilities to receive treatment. However, their permanent residence may be further away. It is possible that some addresses obtained by KCR from health care facility records may be contact addresses (where a patient is staying) rather than permanent addresses, although this may be more relevant for addresses during treatment as opposed to place of residence at the time of diagnosis. This could result in lower-than-expected incidence in outlying areas, impacting observed patterns. However, this also reveals the nature of geographic influences on health in low-resource settings. More work is needed to understand the nature of place of residence in low resource settings, and how residential change, mobility and length of residence may impact exposure and outcome relationships.

## CONCLUSIONS

Cervical cancer is a preventable disease, with available vaccination, screening, and treatment options, but it continues to be diagnosed at high rates among African women and takes many lives. Limited resources should be targeted to areas of greatest need, particularly for a cancer which is commonly the result of a sexually transmitted infection. Mapping cervical cancer incidence with high geographic specificity is feasible in Uganda and reveals clear spatial patterns that can inform resource allocation and support additional research. Future work should identify causal factors associated with observed patterns of cervical cancer incidence, identify spatial patterns of additional cancers, leverage spatiotemporal analysis to examine changes in spatial patterns over time, and utilize maps to inform cancer prevention and control strategies targeting HPV vaccination and cervical cancer screening in Uganda. Future work should also explore residential histories and exposure measurement in relation to cancer outcomes.

This type of approach would be further enhanced by developing a more precise and reliable disease surveillance infrastructure to support decision-making and to better inform the implementation of cancer control strategies and evaluate the impact of programs and policies. Increasing financial and operational support to African cancer registries is essential, as the burden of cancer grows on the continent. Geospatial analysis of African cancer registry data would be optimized through dedicated financial resources; training of cancer registry and/or health facility staff to value, obtain and record geographic data; modification of existing data systems used by LMIC registries (CanReg) to allow for new geographic variables; and alignment with existing census geographies to enable population-based incidence rates and ratios to be calculated. Geocoded, population-based cancer data are essential to an efficient and effective cancer control program and research into the geographic factors influencing cancer outcomes.
